# Test–retest reliability of upper limb robotic exoskeleton assessments in children and youths with brain lesions

**DOI:** 10.1038/s41598-022-20588-8

**Published:** 2022-10-06

**Authors:** Judith V. Graser, Laura Prospero, Monica Liesch, Urs Keller, Hubertus J. A. van Hedel

**Affiliations:** 1 Swiss Children’s Rehab, University Children’s Hospital Zurich, Affoltern am Albis, Switzerland; 2 Children’s Research Centre, University Children’s Hospital Zurich, Zurich, Switzerland; 3 Neuroscience Centre Zurich, University of Zurich and Federal Institute of Technology, Zurich, Switzerland; 4 Research Line Functioning, Participation and Rehabilitation CAPHRI, Department of Epidemiology, Maastricht University, Maastricht, The Netherlands; 5 Department of Health Sciences and Technology, Federal Institute of Technology, Zurich, Switzerland

**Keywords:** Health care, Neurology, Biomedical engineering

## Abstract

In children with congenital or acquired brain lesions, impaired upper limb function can affect independence. Assessing upper limb function is important for planning and evaluating neurorehabilitative interventions. Robotic devices increase measurement-objectivity and enable measuring parameters reflecting more complex motor functions. We investigated the relative and absolute test–retest reliability of assessments to measure upper limb functions in children and adolescents with brain lesions with the exoskeleton ChARMin. Thirty children (9 females, mean age ± SD = 12.5 ± 3.3 years) with congenital brain injuries (n = 15), acquired (n = 14), both (n = 1) and impaired upper limb function participated. They performed the following ChARMin assessments and repeated them within three to seven days: active and passive Range of Motion (ROM), Strength, Resistance to Passive Movement, Quality of Movement, Circle, and Workspace. We calculated the systematic difference, Intraclass Correlation Coefficient (ICC) and Smallest Real Difference (SRD) for each parameter. Six parameters of three assessments showed systematic errors. ICCs ranged from little to very high and SRD values varied considerably. Test–retest reliability and measurement errors ranged widely between the assessments. Systematic differences indicated that random day-to-day variability in performance would be responsible for reduced reliability of those parameters. While it remains debatable whether robot-derived outcomes should replace certain routine assessments (e.g., ROM, strength), we recommend applying certain technology-based assessments also in clinical practice.

Trial registration: This study was registered prospectively at ClinicalTrials.gov (identifier: NCT02443857) on May 14, 2015.

## Introduction

A lesion of the brain, being it congenital or acquired at a later phase in life, might affect motor function, which could negatively influence participation in leisure activities^1^*.* For example, in children with cerebral palsy (CP), the prevalence of upper limb involvement is high and amounts to 83%^2^.

During recent years, rehabilitation technologies have been introduced to compliment conventional therapy interventions. The advantages of these technologies are the high number of movement repetitions, the repeatability with which functions can be practiced and measured, and the goal-oriented training content^3^. As robot-assisted training is usually combined with exergames, motivation^4–6^ and active engagement of the children can be increased playfully^7,8^.

While in research, the evaluation of the effectiveness of such technologies to train upper limb functions in children and youths is increasing (see for example^3,9^) not many studies have investigated the potential of such technologies to assess motor function in a valid, reliable and responsive way. Assessments are important, though, both for planning and monitoring the effects of neurorehabilitative interventions. Measuring functions with rehab-technologies might have various advantages compared to conventional clinical assessments, such as improved objectivity (in contrast to assessments where the performance and scoring are experience or therapist-dependent), precision and accuracy (in contrast to many dichotomous or ordinal scaled clinical assessments), and motivation, when included in game-like scenarios, to remain compliant throughout the assessment.

A robot-aided task proved to be an easy and reliable method to assess proprioceptive sensitivity in typically developing children and young adults^10^. Test–retest reliability of players 5 task assessing upper limb sensorimotor and/or cognitive performance provided by the KINARM robot was evaluated in paediatric hockey players^11^. Intraclass-correlation coefficients (ICCs) varied between 0.06 to 0.91, showing no consistent results^11^. In healthy adults a proprioception assessment (elbow position sense) of the KINARM Exoskeleton Lab showed a fair to good test–retest reliability (ICC = 0.47 (95% CI: 0.14–0.71)) between two sessions^12^. In adults with spinal cord injury, an assessment provided by the Armeo Spring device measuring the upper limb movement workspace showed fair to good reliability^13^. The workspace and several quality of movement metrics measured with the ARMin device showed tendencies to good reliability in adult partients with spinal cord injury^14^.

Nevertheless, the number of studies assessing psychometric properties of assessments evaluating upper limb functions in children with neuromotor disorders.

To fulfil the demands for children with more severely affected upper limb function patients, we developed in a collaboration between the Swiss Children’s Rehab of the University Children’s Hospital Zurich and the Sensorimotor Systems Lab of the Federal Institute of Technology in Zurich the Children Arm Rehabilitation Mechatronic Interface (ChARMin) robotic device^15^ ChARMin is an exoskeleton with drives to support shoulder, elbow, forearm, and wrist movements. It provides virtual reality games to induce engagement and motivation and has seven assessments to quantify various upper limb functions in a standardised manner. Four assessments include functions similar to some conventional therapeutic assessments: (1) active range of motion (aROM) (2) passive range of motion (pROM), (3) isometric strength (Strength), and (4) resistance to passive movement (RPM), as a measure quantifying spasticity. Three other assessments measure more complex movement functions, difficult to assess with routine clinical assessments: (5) quality of goal-directed movements (QoM), (6) dynamic tracking ability of the hand during a circle following task (Circle), and (7) workspace, where we evaluate the maximally reached distances in six movement directions.

In this psychometric study, we aimed to establish the relative and absolute reliability (i.e., the measurement error) of the upper limb ChARMin assessments in children with brain lesions.

## Methods

### Participants

Participants were recruited among the in- and outpatients of the Swiss Children’s Rehab, University Children’s Hospital Zurich, Switzerland.

Inclusion criteria were: (a) age 5 to 18 years, (b) congenital or acquired brain lesion affecting upper limb function, (c) ability to understand and follow test instructions, (d) ability to sit upright for at least 60 min without lateral trunk support, (e) Manual Ability Classification System (MACS) level I-IV (level I: handles objects easily and successfully, level II: handles most objects with somewhat reduced quality and/or speed of achievement, level III: handles objects with difficulty: needs help to prepare and/or modify activities, level IV: handles a limited selection of easily managed objects in adapted situations)^16^.

Exclusion criteria were: (a) severe obesity (i.e., upper limb too large for the robot’s cuffs), (b) fixed upper limb joint contractures, (c) severe spasticity with Modified Ashworth Scale (MAS) > 4^17^, (d) unstable bones or joints, fractures or osteoporosis/osteopenia, (sub-)luxations, (e) upper limb surgery or botulinum toxin injections during the preceding 6 months, (f) skin lesions, (g) implanted devices (e.g. pacemakers, defibrillators), (h) absence of compliance and inability to signal pain or discomfort, (i) severe cognitive deficits, (j) severe visual impairments.

Participants and legal representatives agreed verbally. Legal representatives and participants aged 14 years and older signed written informed consent. The study was approved by the Ethics Committee Zurich (BASEC-Nr. PB_2016-02,450) and the Swiss Agency for Therapeutic Products (Swissmedic reference number: 2015-MD-0009). The study has been performed in accordance with the Declaration of Helsinki.

### Ethical approval

Verbal agreement to participate was obtained from all the participants and their legal representatives. Participants aged 14 years and older and all legal representatives signed written informed consent. The study was approved by the Ethics Committee Zurich (BASEC-Nr. PB_2016-02450) and the Swiss Agency for Therapeutic Products (Swissmedic reference number: 2015-MD-0009). The study has been performed in accordance with the Declaration of Helsinki.

## Materials and procedure

### ChARMin robot

**Figure 1:**
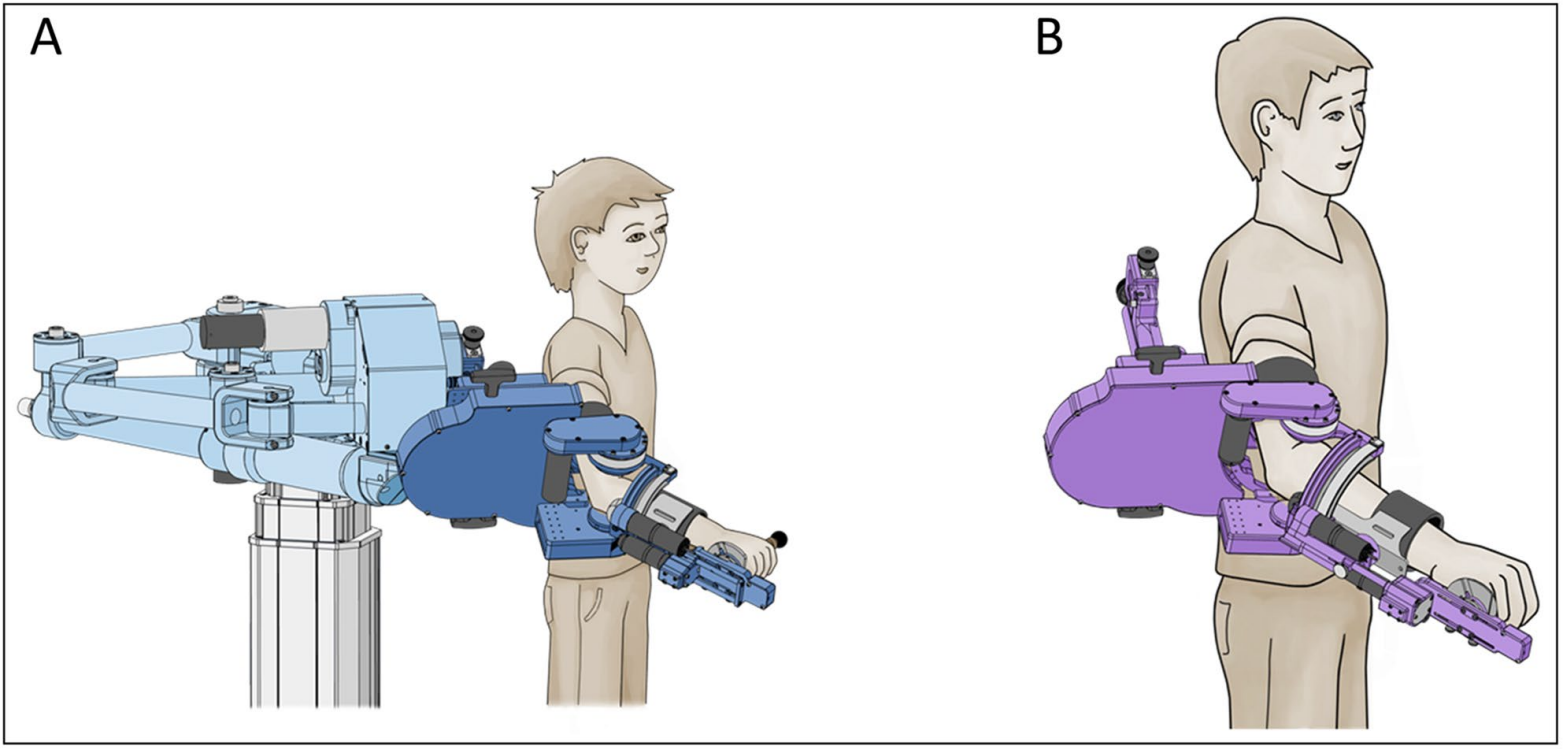
Schematic picture of the ChARMin robot. (**A**) The small distale module (**B**) The large distal module. Courtesy of Susanne Staubli and Urs Keller.

ChARMin is an exoskeleton robot for training upper limb functions^15^. It is attached at the patient’s upper arm and forearm with two hook-and-loop-fastener cuffs. The design is modular. While children can use a smaller distal module, a larger distal module is available for adolescents. The therapist can adjust each module optimally to the individual anthropometrics of each patient (Fig. 1). ChARMin operates with three support modes (non-supported, assist-as-needed, and fully-guided). The robot support can be set between 0 and 100%, which enables training of children with a wide range of impairment severities. ChARMin has six actuated degrees of freedom, which can be moved independently: horizontal abduction/adduction, flexion/extension, and internal rotation/external rotation of the shoulder; flexion/extension of the elbow; pronation/supination of the forearm; and flexion/extension of the wrist. ChARMin’s interface visualizes different games and assessments.

### Procedure

To determine the test–retest reliability of the assessments, the participants attended two measurement sessions, each lasting one hour, scheduled three to seven days apart to obtain stable yet independent measurements. During both sessions, a therapist guided each assessment verbally.

During the whole study procedure, all the children underwent their usual care program, either at the Swiss Children’s Rehab or at their external therapy sites.

*Measurement session 1:* ChARMin was adjusted according to the participant’s anthropometrics of the more affected arm. If both sides were similarly affected, the dominant side was chosen, as training this arm would be clinically more meaningful. After attaching the exoskeleton, the seven assessments were performed in random order, except for the pROM, which was always performed before aROM (since both are included in one ROM assessment) and the RPM. RPM is performed after pROM due to safety reasons, to ensure that the RPM is only moving the joints in the range obtained during pROM.

*Measurement session 2:* All the assessments were repeated in the same order and with the same hard- and software settings as during the first appointment.

### ChARMin assessments

**Figure 2:**
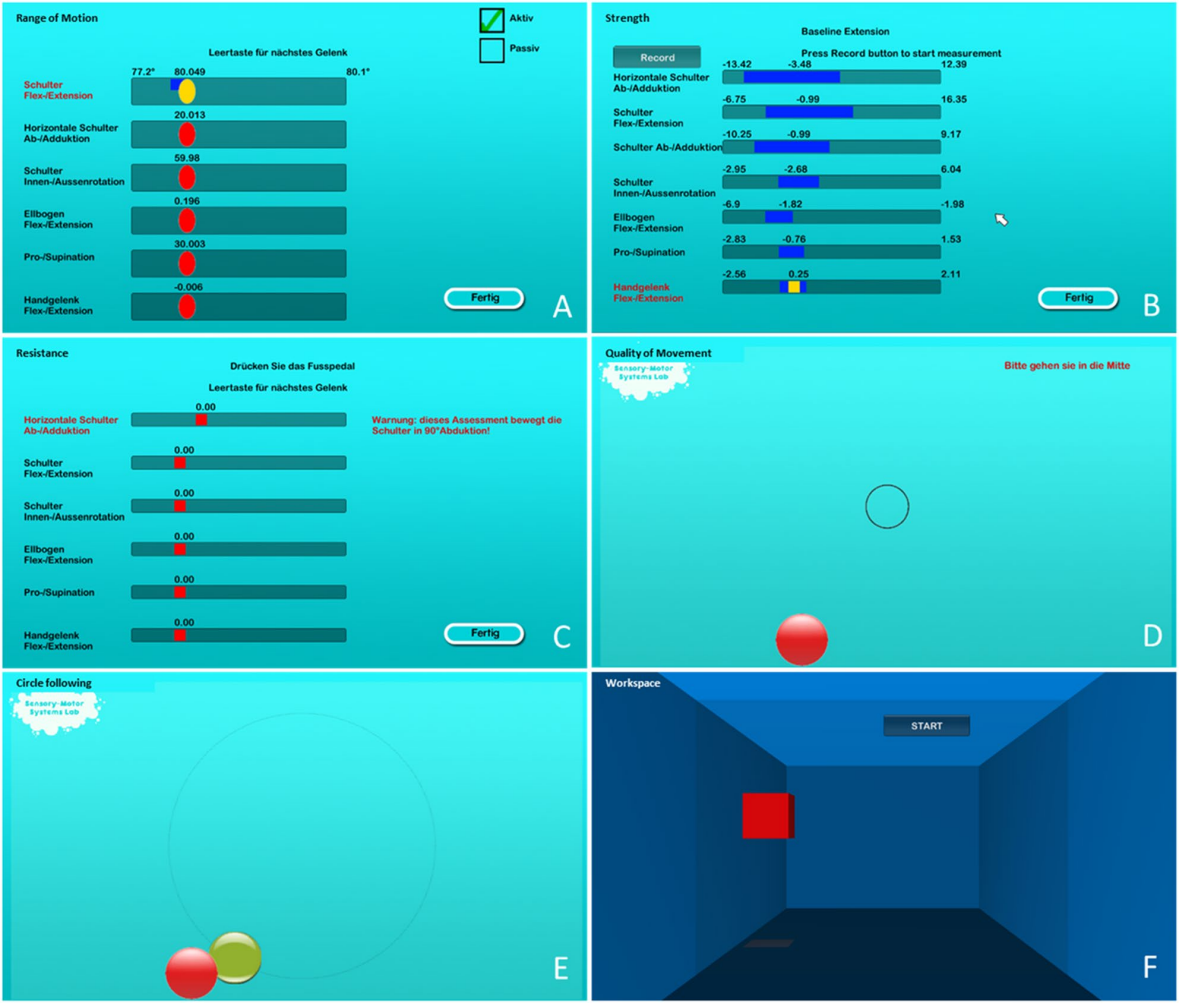
Interfaces of the assessments. (**A**) Active and passive Range of Motion. (**B**) Isometric Strength. (**C**) Resistance to Passive Movement. (**D**) Quality of Movement: eight targets appearing radially around the centre point need to be reached. After each target, the participant has to return to the centre position. (**E**) Circle following: the green ball moves in a circle and the participant is instructed to position the red ball as exactly as possible on the green ball throughout the circular movement. (**F**) Workspace: the participant is instructed to make the virtual room on the screen as large as possible by pushing with the red block against each wall (in forward, backward, left and right direction, respectively), the ceiling upwards and the floor downwards. The block represents the position of the wrist and is steered by moving the arm in the according direction.

ChARMin provides seven assessments to evaluate a wide spectrum of upper limb functions. Figure 2 displays the six assessment interfaces (aROM and pROM have the same interface). We wrote customised algorithms in MATLAB (R2014a, and R2017a, The MathWorks, Inc.) for extracting the assessment parameters. Table 1 provides a more detailed description of the parameters. All the assessments, except for CIRCLE, are based on the ARMin assessments previously presented in^14^. The measurements and calculations of the different assessment scores are identical, except for some smaller adaptations explicitly mentioned in the descriptions below.

**Table 1:** Parameters of the ChARMin assessments.

Assessment	Parameter [unit]	Description
Range of motion (active and passive)	Shoulder horizontal adduction [°]	Maximal range of motion of the shoulder in horizontal adduction
	Shoulder horizontal abduction [°]	Maximal range of motion of the shoulder in horizontal abduction
	Shoulder extension [°]	Maximal range of motion of the shoulder in extension
	Shoulder flexion [°]	Maximal range of motion of the shoulder in flexion
	Shoulder internal rotation [°]	Maximal range of motion of the shoulder in internal rotation
	Shoulder external rotation [°]	Maximal range of motion of the shoulder in external rotation
	Elbow extension [°]	Maximal range of motion of the elbow in extension
	Elbow flexion [°]	Maximal range of motion of the elbow in flexion
	Forearm pronation [°]	Maximal range of motion of the forearm in pronation
	Forearm supination [°]	Maximal range of motion of the forearm in supination
	Wrist flexion [°]	Maximal range of motion of the wrist in flexion
	Wrist extension [°]	Maximal range of motion of the wrist in extension
Strength	Shoulder horizontal abductors [Nm]	Isometric joint torques in the direction of shoulder horizontal abduction
	Shoulder horizontal adductors [Nm]	Isometric joint torques in the direction of shoulder horizontal adduction
	Shoulder extensors [Nm]	Isometric joint torques in the direction of shoulder extension
	Shoulder flexors [Nm]	Isometric joint torques in the direction of shoulder flexion
	Shoulder abductors [Nm]	Isometric joint torques in the direction of shoulder abduction
	Shoulder adductors [Nm]	Isometric joint torques in the direction of shoulder adduction
	Shoulder external rotators [Nm]	Isometric joint torques in the direction of shoulder external rotation
	Shoulder internal rotators [Nm]	Isometric joint torques in the direction of shoulder internal rotation
	Elbow extensors [Nm]	Isometric joint torques in the direction of elbow extension
	Elbow flexors [Nm]	Isometric joint torques in the direction of elbow flexion
	Forearm supinator [Nm]	Isometric joint torques in the direction of forearm supination
	Forearm pronators [Nm]	Isometric joint torques in the direction of forearm pronation
	Wrist extensors [Nm]	Isometric joint torques in the direction of wrist extension
	Wrist flexors [Nm]	Isometric joint torques in the direction of wrist flexion
	Thumb/finger flexors [Nm]	Isometric joint torques in the direction of thumb/finger flexion
Resistance to passive movement (at 60°/s minus resistance occurring at 10°/s)	Against shoulder horizontal abduction [Nm/rad]	Resistance occurring during shoulder horizontal abduction
	Against shoulder horizontal adduction [Nm/rad]	Resistance occurring during shoulder horizontal adduction
	Against shoulder flexion [Nm/rad]	Resistance occurring during shoulder flexion
	Against shoulder extension [Nm/rad]	Resistance occurring during shoulder extension
	Against shoulder external rotation [Nm/rad]	Resistance occurring during shoulder external rotation
	Against shoulder internal rotation [Nm/rad]	Resistance occurring during shoulder internal rotation
	Against elbow extension [Nm/rad]	Resistance occurring during elbow extension
	Against elbow flexion [Nm/rad]	Resistance occurring during elbow flexion
	Against forearm supination [Nm/rad]	Resistance occurring during forearm supination
	Against forearm pronation [Nm/rad]	Resistance occurring during forearm pronation
	Against wrist extension [Nm/rad]	Resistance occurring during wrist extension
	Against wrist flexion [Nm/rad]	Resistance occurring during wrist flexion
Quality of movement (the mean over all movements)	Mean distance-to-path-ratio [unitless]	Length of the trajectory from start/target to the target/start divided by the direct distance between the start and the target position
	Mean standard deviation [m]	Standard deviation of the end-effector position is calculated for the time when the patient’s hand is on the target position
	Mean number of peaks [n speed peaks/distance]	Number of speed peaks normalised to the trajectory distance
	Mean absolute number of peaks	Number of speed peaks
	Mean time [ms]	Difference between the two timestamps when the patient reaches the target and when the patient left the starting position
	Mean reaction time [ms]	Time between the timestamp when the target is shown and the time when the robot end-effector leaves the start/target position
Circle (mean of all the trials)	Mean summed difference [m·s]	Summed difference between the current position and the reference circle
	Mean percentage in front [%]	Percentage of the time that the participant was in front of the reference circle
	Mean ellipse ratio [unitless]	Ratio between the minimal and the maximal radius of the least-squares fitted ellipse
Workspace	Maximum distance lateral [m]	Maximal displacement in direction away from the body, (lateral direction)
	Maximum distance down [m]	Maximal displacement in direction of the bottom wall
	Maximum distance medial [m]	Maximal displacement in direction to the body (medial direction)
	Maximum distance up [m]	Maximal displacement in direction of the top wall
	Maximum distance chest [m]	Maximal displacement in direction towards the chest
	Maximum distance front [m]	Maximal displacement in direction of the front wall
	Volume [m^3^]	Cubic volume of the arm reachable workspace

Parameters obtained from the ChARMin assessments, their unit and the explanation of each parameter.

- ‘aROM’ records the active range of motion [°] of shoulder horizontal abduction and adduction, extension and flexion, and internal and external rotation, elbow flexion and extension, lower arm pronation and supination, and wrist extension and flexion. The child was instructed to move the arm joints actively in the movement directions indicated on the screen.
- ‘pROM’ records the passive range of motion [°] of the same joint movements as listed for aROM. The child should keep the joint relaxed, while the therapist moved the arm joints of the child in the movement directions indicated on the screen.
- ‘Strength’ records the maximum isometric force of muscle groups. The exoskeleton remained in a static position, while the child applied maximal force in the joint direction indicated on the screen. Strength [Nm] was measured for the muscle groups that induce the movements as listed under aROM and pROM. In addition, the strength of the shoulder abductors and adductors, and finger and thumb flexors was measured.
- ‘RPM’ measures the resistance against passive movements analogous to^14^, except that the smaller joint speed was reduced from 30 to 10°/s. After the instruction, the children had to keep their arm relaxed, while the robot moved the child’s arm in each joint direction at a speed of 10°/s and 60°/s. During this movement, the joint torque applied by the force-controlled motors is being recorded. The resistance torque is then calculated by first subtracting the torque that is required to move the joint without the child’s arm from the recording and subsequently extracting the slope of a linear model fitted to the resulting torque–angle characteristics. RPM was assessed for the same joint movements as mentioned for aROM and pROM. We adjusted the signs; a positive value indicated an increase in muscle tone and a negative value a decrease. We subtracted the resistance [Nm/rad] measured at slow velocity (10°/s) from the resistance obtained at high velocity (60°/s) to determine the amount of velocity-dependent increase in muscle tone^18^ in line with the definition of spasticity).
- ‘QoM’ measures the quality of goal-directed movements. The child was asked to move the hand from the centre point on the screen to one of eight different target points appearing one after the other, radially around the centre. After reaching a target, the cursor had to be moved back to the center before a new target appeared. The child performed the task three times. Six parameters, previously described in^14^, quantify aspects of movement quality.
  Movement efficiency is reflected by two parameters: The distance-to-path-ratio [unitless] is the quotient between the direct distance from the start to the target position and the actual length of the path that the patient was taking. The mean standard deviation [m] was calculated from the end-effector position during the 2 s on the target.
  Movement fluency is reflected by the mean number of peaks in the end-effector speed profile [n speed peaks/distance] and mean absolute number of speed peaks [n] as described in^14^.
  Temporal components are reflected by the mean time [ms] to reach the target and the mean reaction time [ms] which is the time from initializing the movement and leaving a threshold circle, 20% wider than the starting position^14^.
  For each parameter, we calculated the average of the three trials.
- During ‘Circle’, the child was asked to follow as accurately as possible a green ball making a circular movement on the screen. The child was instructed to position a red ball (reflecting the wrist position) as precisely as possible on the green ball and follow its circular movement by moving the arm accordingly with the attached exoskeleton. Movement quality was reflected by two parameters: The mean summed difference [m·s] is the integrated difference between the red and the green ball averaged over the executed rounds and the mean ellipse ration [unitless] is the quotient between the smaller and the larger radius of an ellipse fitted to the performed circle on screen., Additionally we calculated a temporal parameter: The mean percentage in front [%] which indicates the percentage in which the red ball from the patient was in front of the green ball with respect to the reference circle on the screen. Similar to QoM, Circle was performed three times and we calculated the average of the three trials for each parameter.
- ‘Workspace’ captures the active workspace of the arm in 3D. The child was instructed to ‘push’ the walls, ceiling, and floor of a virtual room displayed on the screen as far away as possible, making the room as large as possible. The parameters reflect the distance the wrist joint can be moved lateral, medial, up- and downwards, to the chest and to the front [m]. These distances were combined into a seventh parameter, the volumetric workspace [m^3^].
  All the assessments were, whenever possible, adjusted to the abilities of each child (e.g. the speed of the Circle assessment was slowed down from default speed level 5 to speed level 3 if the participant was unable to follow the ball).
  Raw data of Strength and Workspace were measured as positive and negative numbers depending on the direction the force was applied and the spatial direction, respectively. However, for the analysis of these two assessments, absolute values were used. The codes of the evaluation software used to calculate the parameters is available on the figshare database (https://doi.org/10.6084/m9.figshare.9741221).

### Statistical analyses

The statistical analyses were performed with IBM SPSS Statistics 24. We tested data for normal distribution (Shapiro–Wilk-Test). Test–retest reliability was evaluated following a 3-layered approach^19^:

1. *Testing for systematic error:* We applied a paired T-test or Wilcoxon signed-rank test, depending on the data distribution, to test for systematic error between session 1 and 2.
2. *Relative reliability:* We used a two-way mixed model ICC, type absolute agreement^20^. For QoM and Circle we selected the average data option in the model, for the other assessments, we selected single data. Even if data were not normally distributed, ANOVAs are relatively robust to violations of this assumption^21^. ICC values of more than 0.80 were considered as a very high reliability, 0.60–0.79 as a moderately high, 0.40–0.59 as a moderate, and below 0.40 as a low reliability^22^.
3. *Absolute reliability:* The absolute Smallest Real Difference (│SRD│) was calculated based on variance values obtained from the ANOVA table^19^, ^23^ :
  $$\left| {SRD} \right| = {SEM} \times 1.96 \times \sqrt 2 \;$$
  $${\text{where}}\; {SEM} = \sqrt {\left( {s_{residual}^{2} } \right)} \;$$

We calculated │SRD%│, the percentage of the │SRD│ of the Grand Mean (GM; i.e., average of the first and second measurement):

$$\left|SRD\%\right|=\frac{\left|SRD\right|}{GM} \times 100$$

## Results

Nine females and 21 males aged 12.5 ± 3.3 years (mean ± SD) participated. Their height amounted to 139.0 ± 42.3 cm and their weight to 46.9 ± 20.3 kg. Diagnoses were congenital brain injury (n = 15), acquired (n = 14), or both (n = 1). The MACS levels were: level I: n = 8, MACS level II n = 12, MACS level III: n = 8, MACS level IV: n = 1, for one participant the MACS level was not available. Twenty-three participants were inpatients, and seven were outpatients. For 27 participants, the two measurement sessions occurred on the same half of day (i.e. morning or afternoon).

Datasets were excluded for single parameters or assessments if difficulties with compliance on the part of the participants arose (e.g. obvious pushing against the movement of the exoskeleton during the RPM assessment). Furthermore, missing data were produced if the exoskeleton stopped during the procedure mainly due to safety reasons (e.g. resistance against the movement too high). A software bug resulted in a wrong calculation of the data of the Circle assessment leading to the exclusion of all but 14 datasets. The original data used for analysis is available on the figshare database (https://doi.org/10.6084/m9.figshare.9741221).

*Systematic error:* Wilcoxon signed-rank tests showed that six from the 67 parameters differed significantly between the first and second assessment (Table 2). These included the pROM of wrist extension and the RPM against horizontal shoulder adduction. Four of the six parameters of the QoM assessment (i.e. ‘mean distance-to-path-ratio’, ‘mean standard deviation’, ‘mean absolute number of peaks’, ‘and mean time’) indicated that QoM was performed more fluently, with a more direct and faster movement during the second session.

**Figure 3:**
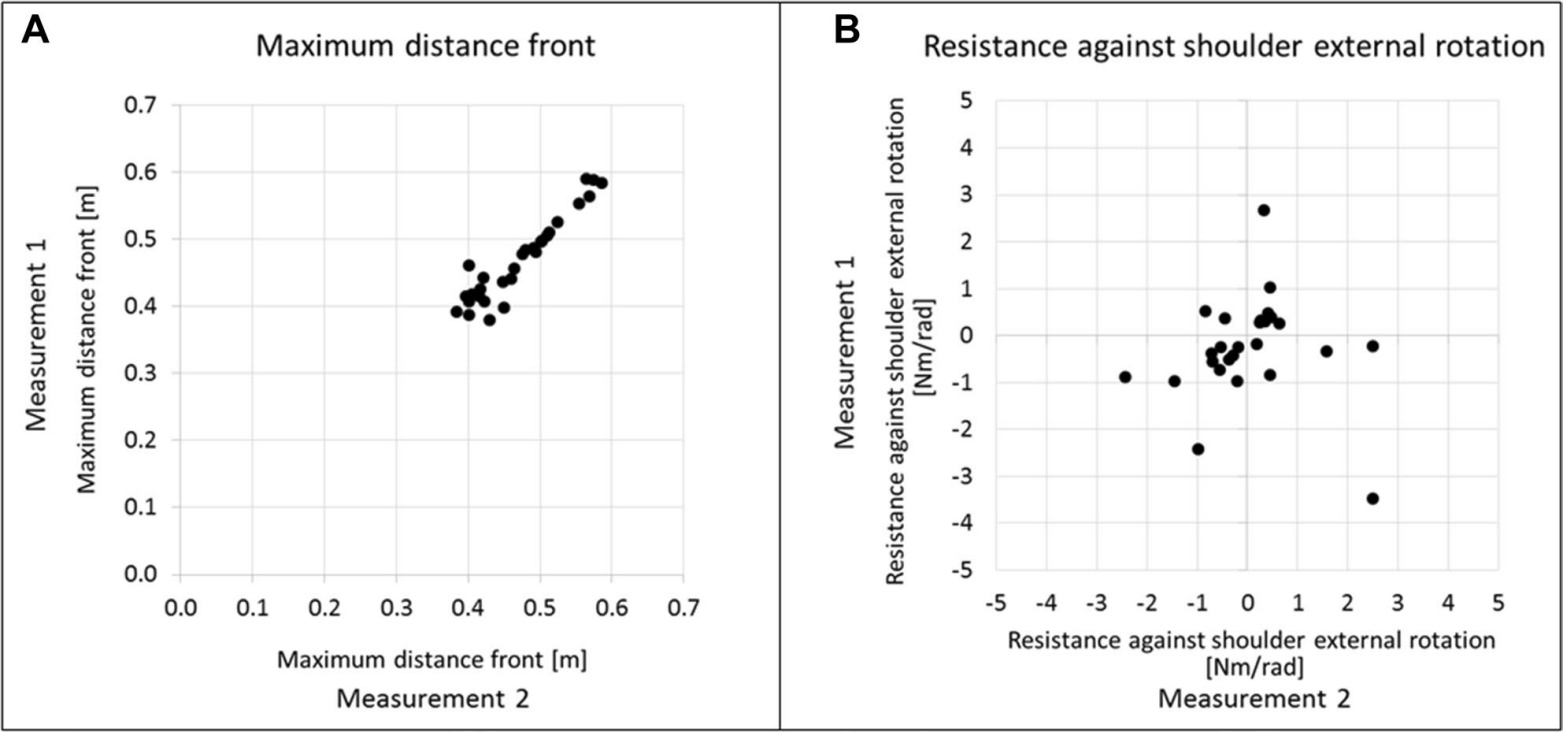
Data distribution of the parameters with the highest and the lowest intraclass correlation coefficients (ICC). (**A**) The parameter ‘maximum distance to front’ of the Workspace assessment which showed the highest ICC (= 0.95). (**B**) The parameter ‘resistance against shoulder external rotation’ of the Resistance to Passive Movement assessment (RPM) which showed the lowest ICC (= − 0.03).

**Figure 4:**
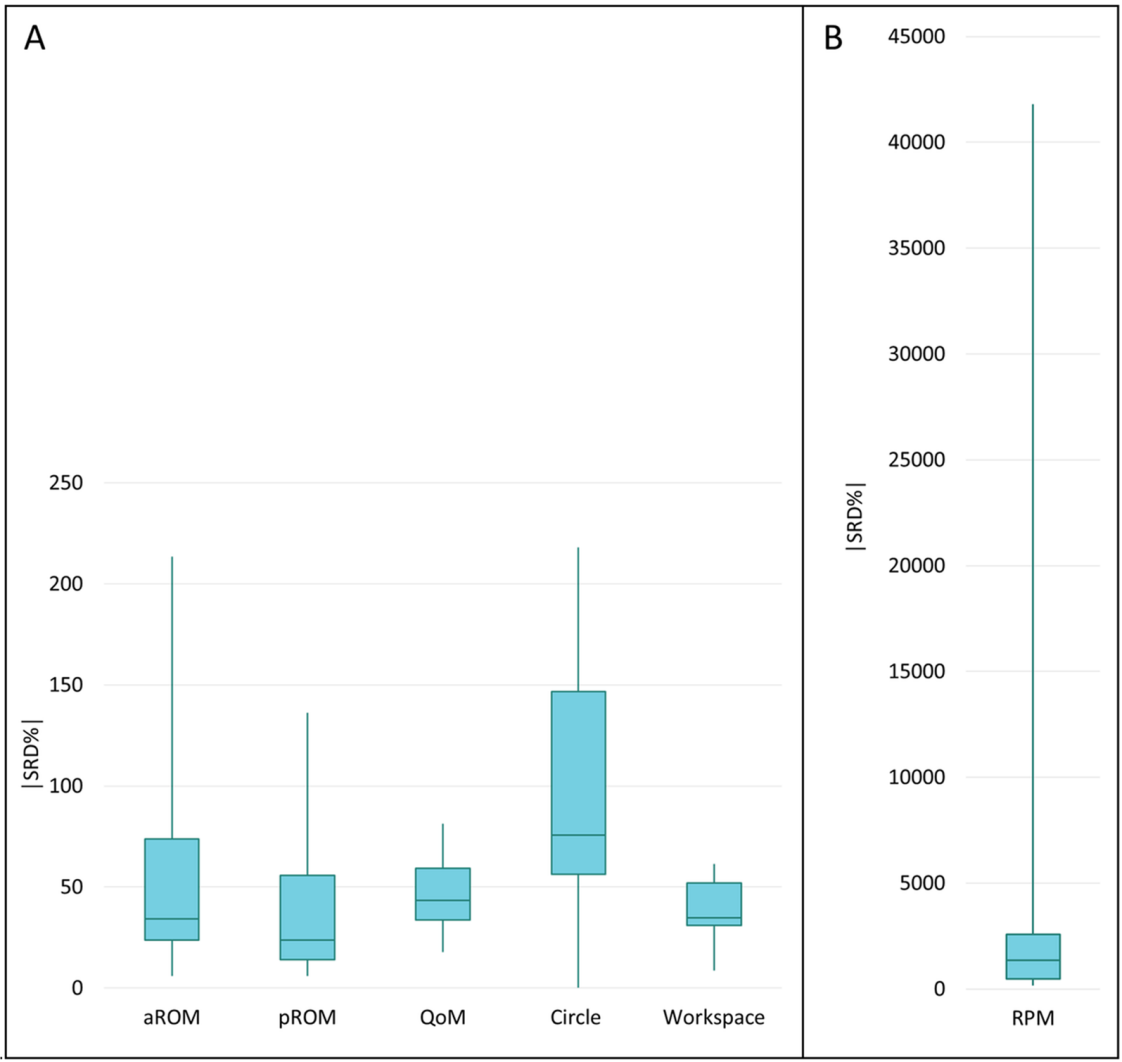
Measurement errors. The box-plots represent the distribution of the smallest real differences as a ratio of the grand means of all the parameters of each assessment. (**A**) Active Range of Motion (aROM), passive Range of Motion (pROM), Strength, Quality of Movement (QoM), Circle, and Workspace assessments. (**B**) Resistance against passive movement (RPM).

1. *Relative reliability:* ICCs ranged from low to very high (Table 2). The highest ICC was obtained for the Workspace parameter ‘maximum distance to front’ (ICC = 0.95, 95% confidence interval (95%CI) [0.89, 0.97]), the lowest ICC in RPM ‘resistance against shoulder external rotation’ (ICC = − 0.03, 95%CI [− 0.41, 0.36]). Figure 3 shows the relationship between the measurements and the distribution of the data of these two parameters.
2. *Absolute reliability:* SRDs for assessment parameters ranged widely within but also between the assessments (Table 2, Fig. 4). The lowest |SRD%| was found for the pROM parameter ‘shoulder extension’ (5.9%) and highest for the RPM parameter ‘resistance against forearm pronation’ (41′810.1%).

**Table 2:** Systematic error, relative and absolute reliability of the parameters of the ChARMin assessments.

Assessment	Parameter [unit]	n datasets in analysis	Systematic error		Relative reliability				Absolute reliability	
			WSR test		95%CI				SRD	|SRD%|
			Median Diff (M1-M2)	p-value	ICC	LB	UB	*p* value		
Active range of motion	Shoulder horizontal adduction [°]	23	− 0.23	0.36	0.19	− 0.25	0.56	0.19	2.88	35.07
	Shoulder horizontal abduction [°]	23	0.34	0.76	0.49	0.09	0.75	0.01	23.84	27.18
	Shoulder extension [°]	27	0.17	0.27	0.70	0.44	0.85	< 0.001	3.18	6.07
	Shoulder flexion [°]	27	0.49	0.07	0.88	0.75	0.94	< 0.001	17.72	15.07
	Shoulder internal rotation [°]	23	− 0.22	0.88	0.54	0.17	0.78	< 0.01	13.14	46.80
	Shoulder external rotation [°]	23	0.43	0.07	0.90	0.79	0.96	< 0.001	15.73	26.45
	Elbow extension [°]	27	8.60	0.12	0.57	0.26	0.78	< 0.001	35.00	213.49
	Elbow flexion [°]	27	− 0.13	0.68	0.82	0.64	0.91	< 0.001	8.50	7.46
	Forearm pronation [°]	25	− 10.12	0.23	0.58	0.26	0.79	< 0.001	34.23	189.10
	Forearm supination [°]	25	37.77	0.06	0.90	0.77	0.95	< 0.001	33.59	88.96
	Wrist flexion [°]	28	− 0.69	0.37	0.66	0.39	0.83	< 0.001	19.32	33.11
	Wrist extension [°]	28	− 11.09	0.39	0.86	0.72	0.93	< 0.001	25.72	68.82
Passive range of motion	Shoulder horizontal adduction [°]	28	− 0.04	0.94	0.55	0.23	0.76	< 0.01	4.56	67.50
	Shoulder horizontal abduction [°]	28	− 0.64	0.12	0.71	0.47	0.85	< 0.001	17.15	20.06
	Shoulder extension [°]	29	0.06	0.54	0.74	0.52	0.87	< 0.001	3.10	5.93
	Shoulder flexion [°]	29	− 0.12	0.30	0.59	0.28	0.78	< 0.001	19.39	15.80
	Shoulder internal rotation [°]	27	− 0.06	0.95	0.01	− 0.38	0.39	0.48	4.29	14.82
	Shoulder external rotation [°]	27	0.04	0.59	0.17	− 0.22	0.51	0.20	7.69	11.70
	Elbow extension [°]	29	0.14	0.13	0.71	0.45	0.86	< 0.001	13.79	136.28
	Elbow flexion [°]	29	− 0.11	0.18	0.14	− 0.23	0.48	0.23	9.98	8.68
	Forearm pronation [°]	26	10.62	0.10	0.54	0.21	0.76	< 0.01	40.29	60.71
	Forearm supination [°]	26	0.22	0.44	0.43	0.06	0.70	0.01	40.28	53.98
	Wrist flexion [°]	29	0.20	0.21	0.54	0.23	0.75	< 0.01	15.45	27.23
	Wrist extension [°]	29	− 10.29	0.03	0.65	0.38	0.82	< 0.001	23.55	48.27
Strength	Shoulder horizontal abductors [Nm]	28	− 3.74	0.30	0.92	0.84	0.96	< 0.001	6.67	58.33
	Shoulder horizontal adductors [Nm]	26	1.58	0.09	0.87	0.71	0.94	< 0.001	8.58	62.12
	Shoulder extensors [Nm]	27	2.54	0.96	0.68	0.42	0.84	< 0.001	16.15	178.76
	Shoulder flexors [Nm]	27	− 3.12	0.18	0.83	0.67	0.92	< 0.001	16.15	68.08
	Shoulder abductors [Nm]	26	− 4.10	0.66	0.34	− 0.05	0.63	0.04	20.94	239.48
	Shoulder adductors [Nm]	27	− 2.26	0.16	0.64	0.35	0.81	< 0.001	17.44	175.02
	Shoulder external rotators [Nm]	27	0.42	0.55	0.90	0.80	0.95	< 0.001	4.72	56.20
	Shoulder internal rotators [Nm]	27	− 0.37	0.09	0.69	0.43	0.84	< 0.001	7.88	119.49
	Elbow extensors [Nm]	27	− 1.52	0.12	0.64	0.36	0.82	< 0.001	9.48	120.02
	Elbow flexors [Nm]	27	− 0.62	0.90	0.89	0.77	0.95	< 0.001	7.13	63.01
	Forearm supinators [Nm]	24	0.15	0.49	0.81	0.61	0.91	< 0.001	1.18	134.90
	Forearm pronators [Nm]	24	− 0.33	0.95	0.61	0.27	0.81	< 0.001	2.11	194.72
	Wrist extensors [Nm]	28	− 0.17	0.73	0.64	0.35	0.82	< 0.001	2.16	156.37
	Wrist flexors [Nm]	28	0.15	0.96	0.60	0.30	0.79	< 0.001	2.96	144.37
	Hand flexors [Nm]	28	0.00	0.40	0.70	0.45	0.85	< 0.001	1.07	132.98
Resistance to passive movement	Against horizontal shoulder abduction [Nm/rad]	26	− 0.13	0.69	0.19	− 0.22	0.54	0.18	4.02	298.54
	Against horizontal shoulder adduction [Nm/rad]	26	0.16	0.01	0.76	0.51	0.89	< 0.001	2.87	526.79
	Against shoulder flexion [Nm/rad]	26	− 2.04	0.29	0.70	0.43	0.85	< 0.001	14.79	672.21
	Against shoulder extension [Nm/rad]	26	1.12	0.18	0.25	− 0.15	0.58	0.10	10.37	1533.00
	Against shoulder external rotation [Nm/ rad]	26	0.26	0.59	− 0.03*	− 0.41	0.36	0.55	3.05	2570.99
	Against shoulder internal rotation [Nm/rad]	26	0.00	0.85	0.72	0.47	0.86	< 0.001	0.87	375.90
	Against elbow extension [Nm/rad]	26	0.05	0.14	0.52	0.18	0.75	< 0.01	3.48	164.34
	Against elbow flexion [Nm/rad]	26	− 0.08	0.42	0.17	− 0.23	0.52	0.20	2.24	1165.18
	Against forearm supination [Nm/rad]	23	− 0.02	0.47	0.70	0.41	0.86	< 0.001	1.15	2634.61
	Against forearm pronation [Nm/rad]	23	0.07	1.00	0.43	0.01	0.71	0.02	0.77	41,810.05
	Against wrist extension [Nm/rad]	26	0.04	0.77	0.22	− 0.19	0.56	0.15	0.81	20,839.08
	Against wrist flexion [Nm/rad]	26	− 0.02	0.29	0.44	0.09	0.70	0.01	0.56	1894.62
Quality of movement	Mean distance-to-path-ratio [unitless]	23	0.12	< 0.01	0.85	0.45	0.95	< 0.001	0.64	35.58
	Mean standard deviation [m]	23	0.00	0.03	0.86	0.64	0.94	< 0.001	0.01	61.73
	Mean number of peaks [n speed peaks/distance]	23	1.05	0.93	0.94	0.86	0.98	< 0.001	4.01	17.71
	Mean absolute number of peaks [n speed peaks]	23	0.96	< 0.01	0.79	0.32	0.92	< 0.001	2.15	33.15
	Mean time [ms]	23	1473.01	< 0.001	0.87	0.40	0.96	< 0.001	2742.99	51.21
	Mean reaction time [ms]	23	259.43	0.10	0.69	0.20	0.88	< 0.001	1032.25	81.26
Circle following	Mean summed difference [m·s]	14	0.01	0.70	0.42	− 0.92	0.82	0.18	1.81	218.06
	Mean percentage in front [%]	14	− 0.23	0.55	0.63	− 0.12	0.88	0.04	39.88	75.54
	Mean ellipse ratio [unitless]	14	− 0.01	0.59	0.73	0.18	0.91	0.01	0.29	37.27
Workspace	Maximum distance lateral [m]	29	− 0.01	0.61	0.84	0.69	0.92	< 0.001	0.11	29.22
	Maximum distance down [m]	29	0.00	0.27	0.71	0.46	0.85	< 0.001	0.10	34.82
	Maximum distance medial [m]	29	0.00	0.30	0.74	0.53	0.87	< 0.001	0.07	32.86
	Maximum distance up [m]	29	− 0.04	0.27	0.92	0.84	0.96	< 0.001	0.12	45.37
	Maximum distance to chest [m]	29	0.00	0.35	0.29	− 0.07	0.59	0.06	0.13	58.58
	Maximum distance to front [m]	29	0.00	0.75	0.95	0.89	0.97	< 0.001	0.04	8.58
	Volume [m^3^]	29	− 0.01	0.09	0.91	0.82	0.96	< 0.001	0.06	61.50

Results of the Wilcoxon signed-rank test (WSR test) investigating the systematic error. Displayed are all the 67 parameters of the assessments. Intraclass Correlation Coefficients of Quality of Movement and Circle assessment are based on average measures. Intraclass correlation coefficients of active and passive Range Of Motion, Strength, Resistance to Passive Movement and Workspace are based on single measures.

Abbreviations: Diff. = difference; M1 = median of the first measurement; M2 = median of the second measurement; ICC = intraclass correlation coefficient; 95%CI = 95% confidence interval; LB = lower bound; UB = upper bound; SRD = smallest real difference; SRD% = smallest real difference/grand mean × 100.

*A negative ICC is referred to as a bad or unfortunate estimate, possibly occurring by chance, especially with a small sample size ^39^.

**Figure 5:**
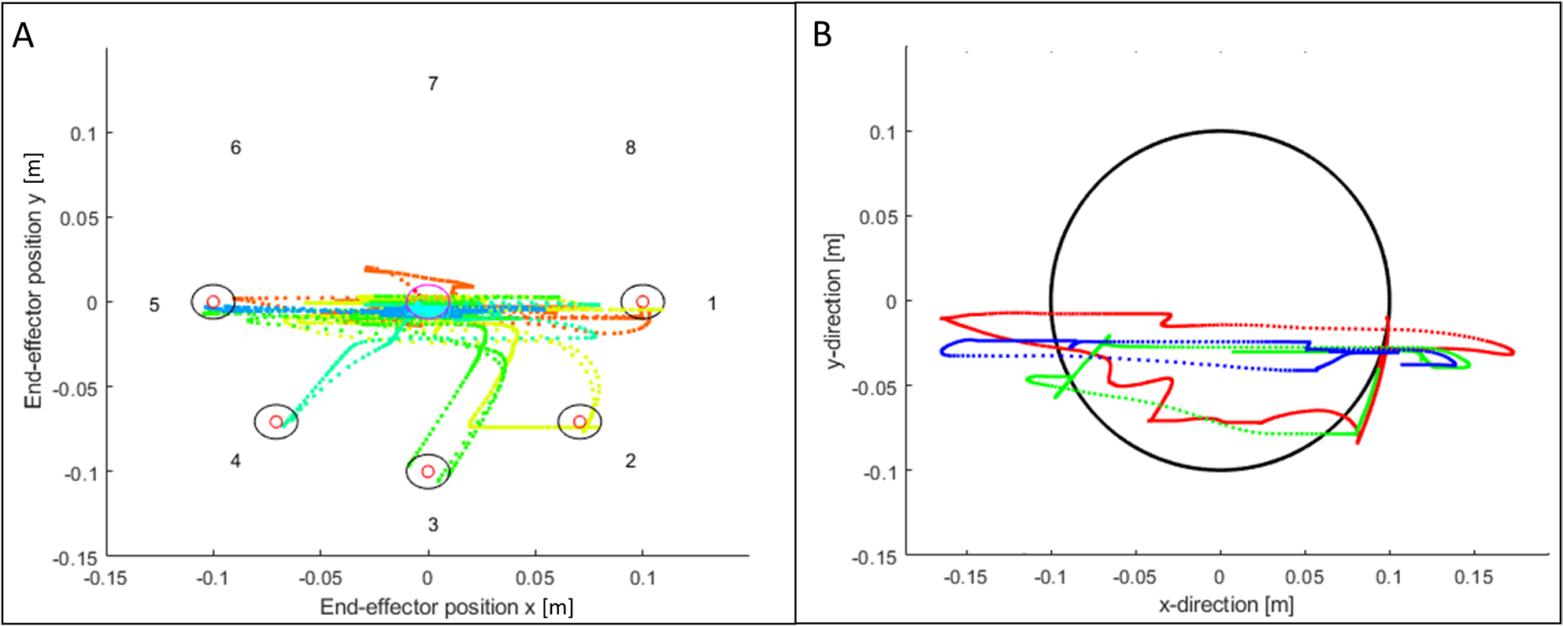
Examples of trajectories of Quality of Movement and Circle assessmentsThe trajectories were obtained from data of an adolescent participant with acquired hemiparesis and a MACS level III who had difficulties in moving the arm upwards against gravity. (**A**) Quality of Movement assessment: Paths for the movements from the targets to the centre point. Upper targets were not reached. (**B**) Circle assessment: Paths of the three rounds of tracking the ball moving in a circle. The upper part of the circle was not reached. Red line = round one, green line = round two, blue line = round three. The excursion of the movement becomes smaller with each round.

Additional scatterplots indicating the relationships between the first and second measurements and the distribution of the data of each assessment and parameter (see Supplementary Information files 1 to 6).

## Discussion

We evaluated the relative and absolute test–retest reliability of many parameters included in seven assessments integrated in the ChARMin robot in children with congenital or acquired brain lesions. Unlike clinical assessments requiring a therapist’s opinion, ChARMin measures accurately and the parameters were derived from standardised software algorithms. We found a systematic difference between the first and second measurement for a few parameters and we will discuss this in more detail later. Still, analyses of relative and absolute reliability of the other parameters showed large differences between the parameters and assessments. These findings indicate that for the parameters with poor reliability results, most likely the patients have introduced random variability between two measurements, for example, due to day-to-day fluctuations in motor functioning, fatigue, attention, or compliance. Such findings are important when rehabilitation specialists discuss whether objective technology-based outcomes should complement or even replace clinical routine assessments.

### aROM and pROM

Our results indicated that test–retest reliability of aROM and pROM varied largely between the different joints and movement directions. While most aROM and pROM ICCs varied between moderate and very high, reliability for some joint directions was low. The conventional method to measure ROM is using a manual goniometer. Concerning the aROM, one study evaluated the intra-rater reliability of goniometry in children after forearm fractures^24^. ICCs ranged from 0.73 to 0.97 for pronation and from 0.80 to 0.97 for supination in different age groups^24^. While the result for supination from our study (ICC = 0.90) is in line with these results, we found poorer pronation reliability results (ICC = 0.58). Differences could be explained by factors such as the patient population (impairments, cognitive abilities influencing the test performance), or the number of movement repetitions. While Colaris et al^24^ calculated means over three measurements, making numbers more stable, our results were based on one ROM measurement only. These factors could also underlie the better absolute errors reported by Colaris et al^24^, as the SRD ranged from 4 to 9 degrees for pronation and 5 to 9 degrees for supination.

Test–retest reliability of pROM was investigated in 23 children with CP aged around 10 years 6 months^25^. Tests-retest reliability was good; the ICC for pROM elbow extension was 0.94, 95%CI [0.86, 0.97], for forearm supination 0.81, 95%CI [0.61, 0.91], and for wrist extension 0.88, 95%CI [0.74–0.95]. Our reliability results were lower (elbow extension: ICC = 0.71; forearm supination: ICC = 0.43; and wrist extension: ICC = 0.65). Differences in results might be explained by the test–retest time interval (≥ 3 days in our study versus 1 h in the study by Klingels et al^25^ or the study participants (while our participants showed moderate to major impairments, Klingels et al^25^ did not evaluate should flexion and abduction because only three or four participants showed impairments). Interestingly, inter-rater reliability results from the same study (n = 30 participants) were poorer compared to the test–retest reliability results (elbow extension: ICC = 0.69; forearm supination: ICC = 0.73; and wrist extension: ICC = 0.48) showing the dependency on the rater for the clinical assessment.

In a robotic pROM assessment in healthy adults, ICCs for wrist flexion were 0.97–0.98 and for wrist extension 0.87–0.95, while the SRD% varied between 9.9 and 19.6% for both movement directions^26^. Reliability of our data is considerably lower, which can be explained by the different groups (healthy adults versus children with brain lesions) or differences between the technologies. We discuss some technical limitations of ChARMin in the limitation section.

### Strength

Our results indicate moderately high to very high reliability for the muscle strength parameters, except for shoulder abduction (low reliability). Muscle strength is conventionally measured with the manual muscle test^27^. In children with CP, this assessment showed a test–retest reliability with ICCs from 0.69, 95%CI [0.40, 0.85] for the shoulder abductors to 0.98, 95%CI [0.95, 0.99] for forearm supinators^25^. Finger and thumb flexion (or grip strength) can be measured with the manual muscle test but is frequently assessed with the Jamar dynamometer. While ChARMin could assess grip strength with moderately high reliability, grip strength dynamometry shows excellent reliability in children with CP (test–retest: ICC = 0.96, 95%CI [0.90, 0.98]; and inter-rater: ICC = 0.95, 95%CI [0.89, 0.97] ^25^. In our study, the ICCs for most strength assessments were lower compared to Klingels et al.^25^, which could, again, be explained by the longer test–retest interval and different patient characteristics.

Compared to the other ChARMin assessments, the relative reliability of Strength was rather good, but the absolute reliability showed partly large measurement errors (Table 2, Supplementary Information file 2).

### RPM

The reliability of the RPM parameters varied between low and moderately high. One study evaluated test–retest reliability of the MAS, the most commonly used spasticity assessment, in children with CP and found ICCs varying between 0.70, 95%CI [0.42, 0.86] for shoulder adductors and 0.85, 95%CI [0.69, 0.93] for elbow flexors^25^. Another study evaluating the Ashworth Scale in patients with upper motor neuron syndrome concluded that it is not valid and reliable enough to measure spasticity^28^. Indeed, spasticity can fluctuate making it difficult to assess it reliably. An objective measure quantifying spasticity better would be valuable, as many interventions, e.g. in children with CP, focus on reducing muscle tone and preventing joint contractures.

Spasticity is defined as an increase in velocity-dependent stretch reflex^29^. It is recommended to stretch the muscle from one end position to the other within one second^17^. For the elbow flexors, for example, this would mean a speed of about 180°/s if the patient has no joint contracture. The RPM assessment provided by ChARMin moves slower, i.e., 60°/s at fast speed, for safety reasons. While we found in general higher levels of resistance during the faster movements, it remains unclear whether a faster high-velocity condition would have resulted in results that are more reliable. Also, again for safety reasons, ChARMin might not have moved each joint through the full pROM. Hence, end-range movements are not tested which prevents detecting a catch and release phenomenon as it is done with the MAS^17^.

### QoM

Five of the six QoM parameters showed very high reliability, one moderately high. Yet, four parameters improved significantly from the first to the second test occasion.

Measuring quality of movement is challenging. It starts with the question about what aspects of movement reflect movement quality. In a qualitative study, physiotherapists were interviewed about the phenomenon “movement quality”^30^. Therapists mentioned, among others, “the movement characteristic of path and form in movement” and “the movement characteristics of flow, elasticity, and rhythm”^30^, confirming most of the parameters that we had included in our QoM assessment.

In our study, participants seemed to perform the QoM with a more fluent, more direct, and faster movement at the second session indicating familiarization to the assessment. However, statistically, we did not correct for multiple comparisons and false positive results might have occurred. When dividing the *p* value of 0.05 by 67 (number of comparisons) the corrected p-valued would be 0.0007 and only the parameter ‘mean time’ would still be significant. Nevertheless, if the QoM assessment would be used to measure change in movement quality due to an intervention, this familiarization aspect should be taken into account. Parameters of the QoM assessment measured with the precursor device of ChARMin have shown a similar tendency to systematic error in healthy adults^14^.

When a similar point-to-point reaching task was performed by healthy adults with the end-effector robot MIT-Manus in the horizontal plane, no systematic error was found when evaluating data from six measurement sessions on three days (two sessions per day) over two to three weeks^31^. The different reliability results might be explained by differences in task performance (i.e. vertical plane versus horizontal plane, which might reduce the dependency on muscle strength), and the study design.

We noted during the QoM assessment (i.e. reaching eight targets and repeating the procedure three times) that some children became less motivated during the procedure. Some children, with limited strength of antigravity muscles, had difficulties reaching the upper targets (Fig. 5), but adding physical support from ChARMin to reach the higher targets made it more difficult to reach the lower targets as the participants had to push the device downwards. We would recommend including such an assessment in an exergame scenario making it more suitable and interesting for children. As QoM parameters reflecting movement quality, which is difficult to obtain with current routinely applied clinical assessments, we consider this assessment of great interest for upper limb paediatric rehabilitation. Particularly the parameter ‘number of speed peaks normalised to the actual path’ reflecting movement fluency showed excellent reliability and no systematic change making it reliable enough to serve as an outcome parameter.

### Circle

The parameters of the Circle assessment reflecting movement quality showed moderate to moderately high reliability. The MIT-Manus also provides a similar assessment that seems reliable in healthy adults^31^.

Similar to the QoM, it was difficult to reach the full circle for children who had difficulties moving the arm against gravity (Fig. 5). Unfortunately, the processing software revealed a bug, which lead to the exclusion of multiple datasets. The interpretation of the results obtained from 14 datasets is limited. Generally, and also similarly to the QoM, the Circle assessments would be able to measure clinically relevant parameters reflecting more qualitative aspects of movement. We think a parameter like the “mean ellipse ratio”, which informs about the “movement characteristic: seeing the path and form in movement”^30^ could be of interest to quantify movement accuracy, as it showed a moderately high test–retest reliability.

### Workspace

The Workspace assessment provided parameters showing moderately high to very high test–retest reliability, with the exception of the “maximum distance to chest” parameter. We assume that the low reliability ICC coefficient found for the parameter ‘maximum distance to chest’ can be explained by the relatively fixed endpoint (reaching the chest), resulting in a low *between-subjects* variability.

Reliably quantifying reaching distance and workspace volume can be of interest, because it is likely more relevant for various ADL as compared to single joint ROM parameters. We are unaware of a conventional clinical assessment that would provide this information in such an easily applicable way. The reliability of the workspace volume has been evaluated with the ARMEO Spring device in adult patients with neurological deficits and healthy participants^13^. Intra-rater reliability showed ICCs from 0.75 to 0.86 for healthy adults in different seating positions and an ICC of 0.86 for eight adults with neurological upper limb impairments sitting in the chair used at the current phase of rehabilitation^13^. These results are also good and slightly below the ICC value obtained in this study. Some of the differences could be caused by the different groups of participants and while we evaluated the workspace volume for one side, they included the workspace volumes of both sides in the same analysis.

One limitation of this assessment was that some participants were not able to reach the centre position, which is needed to start the assessment, without support. While the therapist had to support the child to reach the starting position, this did not affect the data.

Due to the easiness of application and its potential relevance for ADL, we think measuring reaching distance sideways, up- and downwards, and forwards, as well as the workspace volume seem practical and reliable outcome parameters in children with brain lesions.

### Clinical implications

Major advantages of the ChARMin and other robot-assisted devices are the objectivity of the measurements and the quantification of parameters assessing more complex aspects of upper limb motor functions, which are usually not covered by conventional assessments. However, according to our experience, it should carefully be pondered when the use of a device like ChARMin for assessing such functions is reasonable. Adapting the exoskeleton to the anthropometry of the child and creating a user profile is quite time-consuming. While this is not more time-consuming for children who train with the device, it seems not justifiable to apply ChARMin for assessments only, particularly if one is interested in assessing functions that can be assessed at least as reliable with conventional assessments like aROM, pROM, strength, or spasticity. Furthermore, the application of devices in rehabilitation is considered a continuum, in terms of starting with a device that can provide physical support and switching to another device when functions improve^32,33^. This is particularly relevant for patients with acquired brain lesions who show considerable recovery during the first six months post injury. To ensure that during the whole recovery and/or rehabilitation process, changes in function can be assessed longitudinally, assessments such as included in ChARMin, could be complemented with conventional assessments that can be applied continuously during these processes, such as the Melbourne Assessment 2^34^ or the Assisted Hand Assessment^35^. As another option, more practical technologies need to be developed that would allow valid, reliable and responsive objective measurement throughout recovery and/or rehabilitation and could also assess the more complex motor functions, as investigated here in the QoM or Circle assessments, or the Workspace assessments. To keep particularly young patients motivated and engaged, such technologies could use exergame like scenarios, enabling to record outcome parameters while playing.

### Limitations

We included participants with congenital or acquired brain injuries affecting upper limb function. Two participants had besides the brain lesions several comorbidities, which could have influenced upper limb impairments additionally. While this increased the heterogeneity of the study sample, it reflects the population of children undergoing upper limb neurorehabilitation.

While we calculated the SRD values, both absolute and relative, it remains difficult to interpret the magnitudes of these values before we have investigated the responsiveness and the minimal clinically important difference of the parameters. Having mentioned that, particularly the high relative SRD values of some parameters indicate that the huge variability will make it very difficult for these parameters to be considered responsive.

The ROM provided by ChARMin is large enough to train daily life relevant movements. However, ChARMin has mechanically limited ROM due to safety reasons. Therefore, measuring full ROM for horizontal shoulder adduction and abduction, shoulder flexion and extension, shoulder internal and external rotation and elbow flexion is not possible. As this might have limited the variability *between* participants, it might have affected the ICC values negatively, as these express the amount of between-subject variability divided by the sum of between-subject and within-subject systematic and random variability.

Children with CP have difficulties in generating maximal strength^36^, but also in relaxing muscles which leads to difficulties in activities of daily life^37^. The acknowledgement of both, muscle weakness and stiffness is relevant for the pROM, Strength, and RPM assessments. For example, when performing isometric strength measurements, we had to subtract a baseline measurement reflecting ‘no force’, i.e., where the participants had to relax. Based on our experience, we would recommend that such a ‘baseline’ should not be measured *before* the maximal isometric strength measurement, but *after, as* participants seem better able to relax. Relaxing the muscles is also important to quantify resistance to passive movement (i.e. spasticity) in the RPM assessment. The child’s arm is attached to a moving exoskeleton, which is unfamiliar and some children experienced it as frightening. This makes it difficult to determine whether we actually assessed increased muscle tone during the faster movement, or increased ‘active’ resistance during the faster perhaps somewhat more uncomfortable movement in the robot. A general issue when comparing resistance against slow and fast movements to quantify spasticity is that it is difficult to differentiate between a velocity-depended increase in muscle tone and passive structures (e.g. joint capsule, ligaments) as origin of the resistance. Muscles and passive soft tissues are viscoelastic, meaning that the resistance due to passive stretching is velocity dependent and increases with the velocity of a stretch, as spasticity does^38^.

An additional issue with the Strength assessment was that some participants with relative good muscle strength could generate enough torque to activate the safety software which stopped the device. Indeed, ChARMin was initially developed for patients with major impairments. This observation shows that it might be difficult to assess isometric strength on the long-term reliably in patients who recover well.

Some technical problems (e.g. bugs in software), compliance issues of the participants, and the inability to perform some assessments led to missing data. ChARMin was developed for children with more severe impairments. However, our study showed that participants already require certain abilities (e.g. move the shoulder against gravity, able to relax the arm) to perform the assessments. ChARMin is a unique specimen and not commercially available so far. We hope that our findings might be useful for rehabilitation engineers and manufacturers to improve the development and implementation of assessments in rehabilitation technologies for the use in clinical practice.

## Conclusions

Performing assessments reliably with an objective and accurate arm exoskeleton device in children with brain lesions proved much more challenging as expected. Relative and absolute test–retest reliability of 67 parameters included in seven assessments evaluating upper limb functions provided by the ChARMin varied considerably from low to very high between and within the assessments. While we found some assessments promising because they provided novel quantitative reliable information on motor function, we noted difficulties with other assessments that need to be improved before being applied in neurorehabilitation.

## Supplementary Information

Supplementary Information 1.

Supplementary Information 2.

Supplementary Information 3.

Supplementary Information 4.

Supplementary Information 5.

Supplementary Information 6.

## Data Availability

The datasets generated and analysed used for analyses during the current study are available in the figshare repository, https://figshare.com/articles/journal_contribution/Reliability_ChARMin_assessments/9741221.

## Code availability (software application or custom code)

The evaluation software (Matlab codes) used for analyses during the current study is available in the figshare repository, https://figshare.com/articles/journal_contribution/Reliability_ChARMin_assessments/9741221.
