# Supplementary material for: Test–retest reliability of upper limb robotic exoskeleton assessments in children and youths with brain lesions
Source: Sci Rep. 2022 Oct 6;12:16685. doi: 10.1038/s41598-022-20588-8 (PMC9537308; doi:10.1038/s41598-022-20588-8)
Supplement: Supplementary file 1 — Supplementary Information 1. [file 41598_2022_20588_MOESM1_ESM.pdf]

Supplementary information file 1

Distribution of data of each parameter of range of movement assessments provided by ChARMin

A) Active Range of Motion

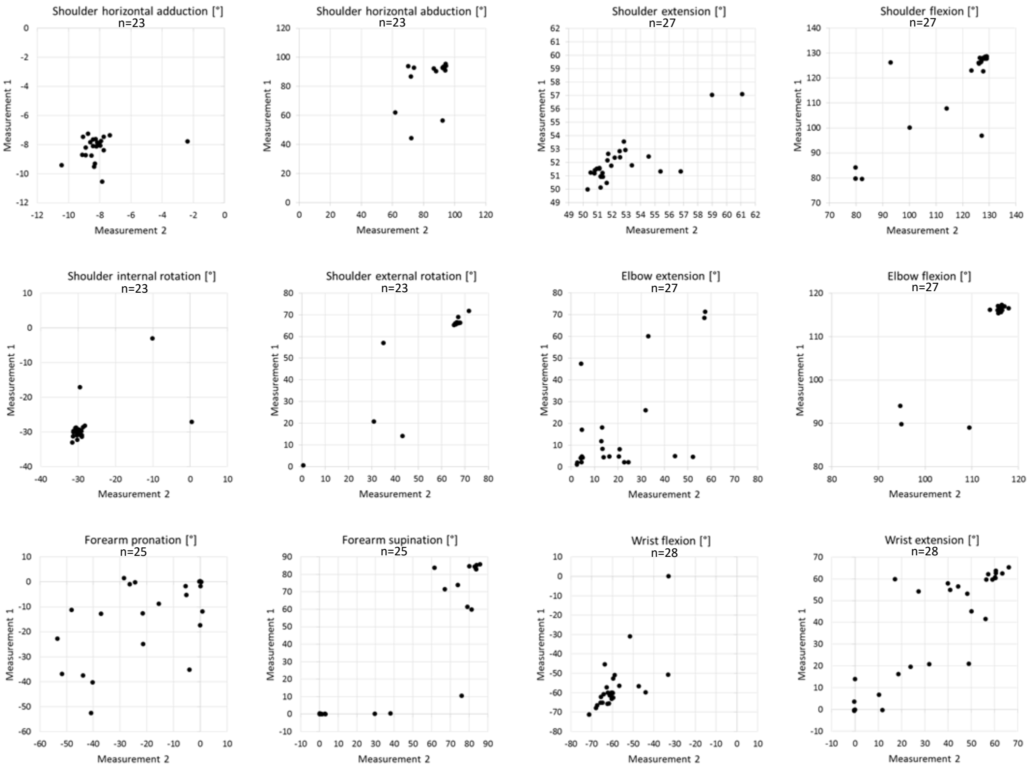

B) Passive Range of Motion

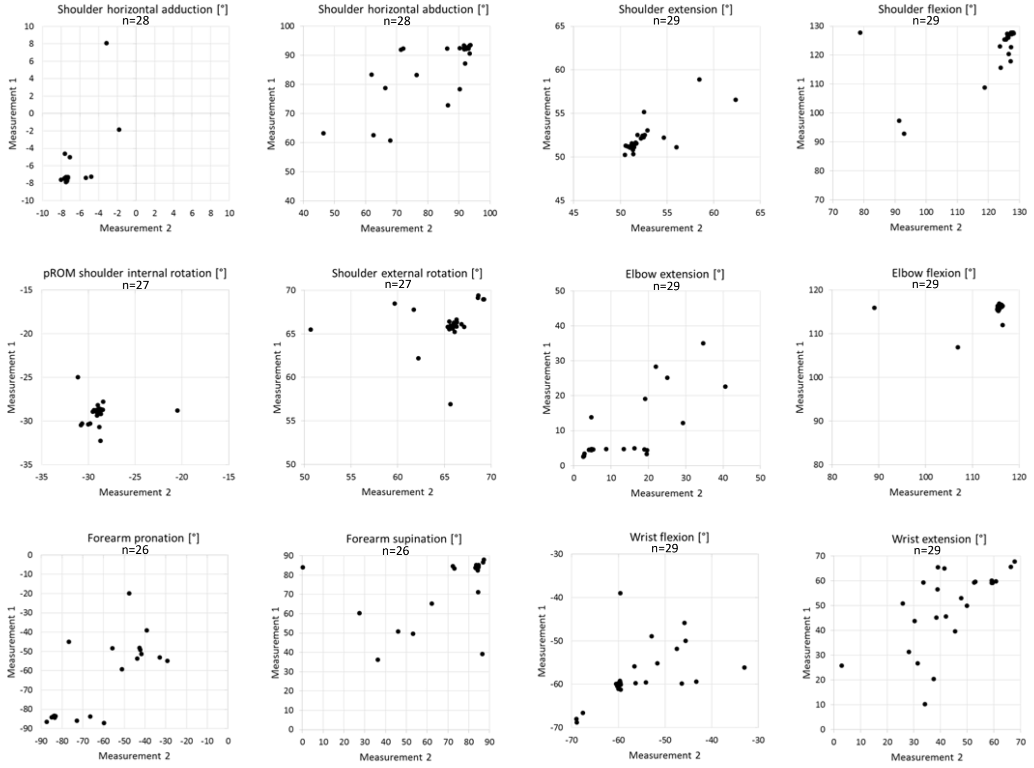

Displayed are the data of each parameter of A) active Range of Motion (aROM) assessment and B) passive Range of Motion assessment (pROM). Each movement axis contains two movement directions. Movement directions are displayed as negative or positive numbers, respectively: shoulder horizontal adduction and abduction, shoulder extension and flexion, shoulder internal rotation and external rotation, elbow extension and flexion, forearm supination and pronation, wrist flexion and extension. The respective end-position of the joint angle is displayed in degrees (°). The X-axis represents the second measurement, the Y-axis represents the first measurement.
