# Supplementary material for: Test–retest reliability of upper limb robotic exoskeleton assessments in children and youths with brain lesions
Source: Sci Rep. 2022 Oct 6;12:16685. doi: 10.1038/s41598-022-20588-8 (PMC9537308; doi:10.1038/s41598-022-20588-8)
Supplement: Supplementary file 2 — Supplementary Information 2. [file 41598_2022_20588_MOESM2_ESM.pdf]

Supplementary information file 2

Distribution of the data of each parameter obtained from the Strength assessment

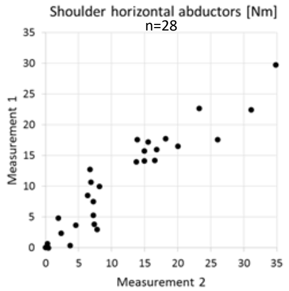

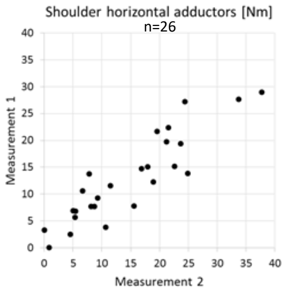

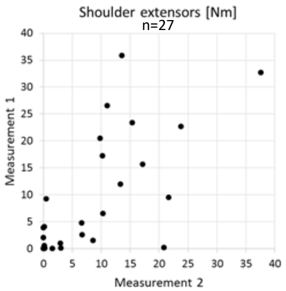

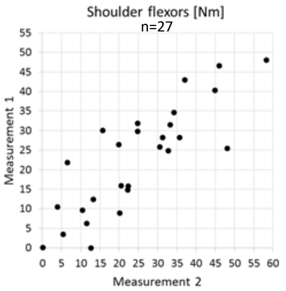

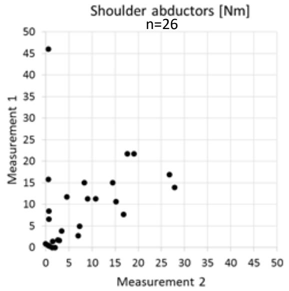

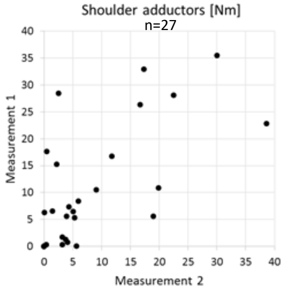

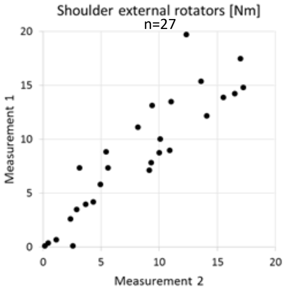

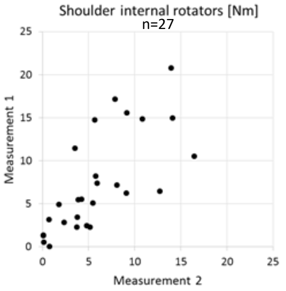

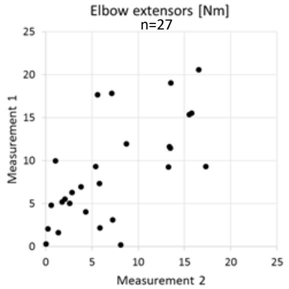

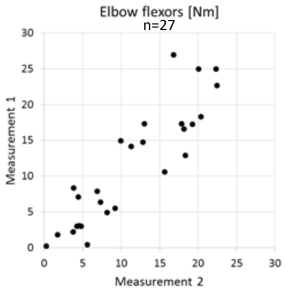

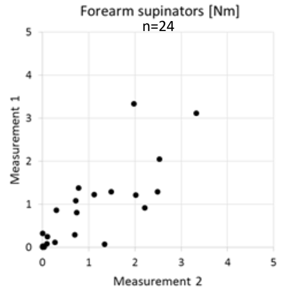

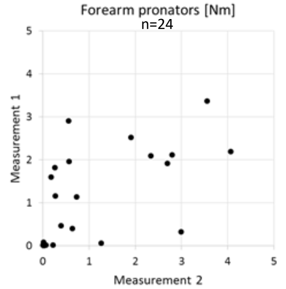

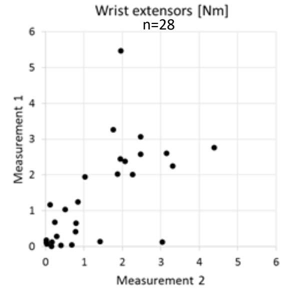

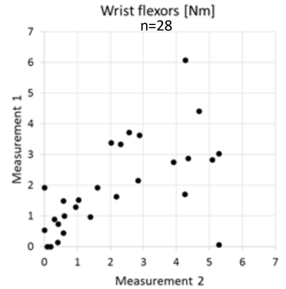

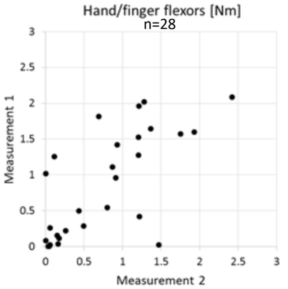

Displayed are the absolute data of each parameter the Strength assessment in Newton meter (Nm): Strength of the shoulder horizontal abductors and adductors, shoulder extensors and flexors, shoulder abductors and adductors, shoulder external rotators and internal rotators, elbow extensors and flexors, forearm supinators and pronators, wrist extensors and flexors and hand/finger flexors. The X-axis represents the second measurement, the Y-axis represents the first measurement.
