# Supplementary material for: Test–retest reliability of upper limb robotic exoskeleton assessments in children and youths with brain lesions
Source: Sci Rep. 2022 Oct 6;12:16685. doi: 10.1038/s41598-022-20588-8 (PMC9537308; doi:10.1038/s41598-022-20588-8)
Supplement: Supplementary file 3 — Supplementary Information 3. [file 41598_2022_20588_MOESM3_ESM.pdf]

Supplementary information file 3
Distribution of the data of each parameter obtained from the Resistance to Passive Movement assessment

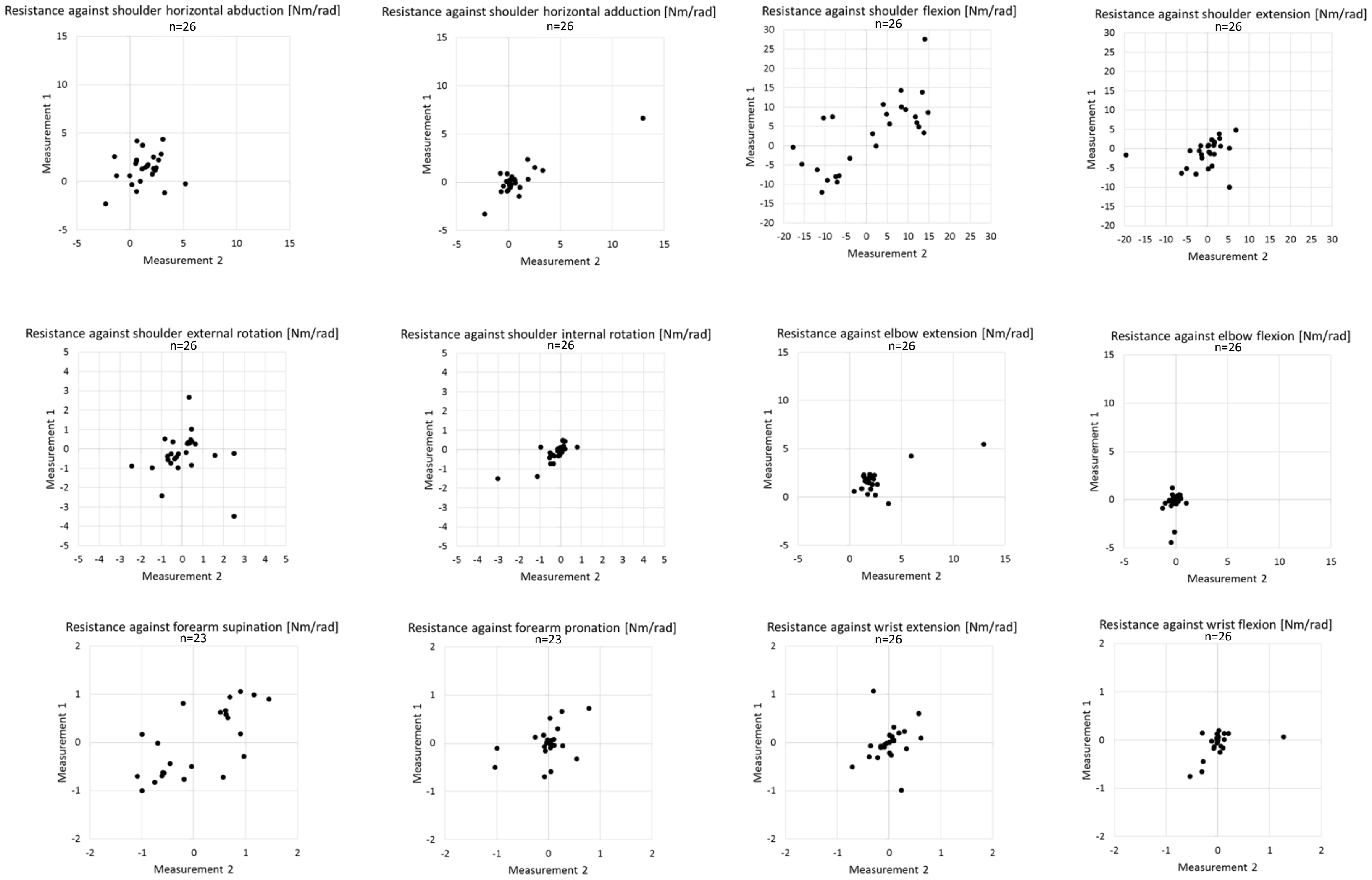

Displayed are the data of each parameter the Resistance to Passive Movement (RPM) assessment in Newton meter per degrees (Nm/rad). The plots show the respective differences between the resistance against passive movements with a speed of 60 seconds per degree and the resistance against passive movements with a speed of 10 seconds per degree of the following movement directions: resistance against horizontal shoulder abduction and adduction, shoulder flexion and extension, shoulder external and internal rotation, elbow extension and flexion, forearm supination and pronation, wrist extension and flexion. The X-axis represents the second measurement, the Y-axis represents the first measurement.
