# Supplementary material for: Test–retest reliability of upper limb robotic exoskeleton assessments in children and youths with brain lesions
Source: Sci Rep. 2022 Oct 6;12:16685. doi: 10.1038/s41598-022-20588-8 (PMC9537308; doi:10.1038/s41598-022-20588-8)
Supplement: Supplementary file 4 — Supplementary Information 4. [file 41598_2022_20588_MOESM4_ESM.pdf]

Supplementary information file 4
Distribution of the data of each parameter obtained from the Quality of Movement assessment

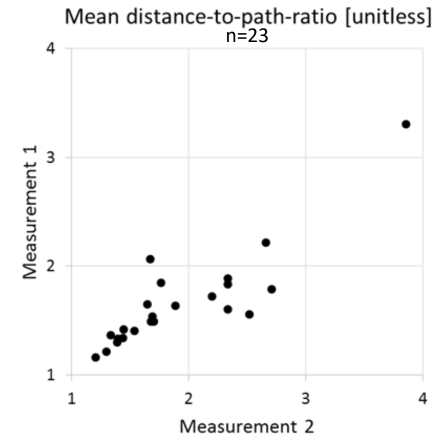

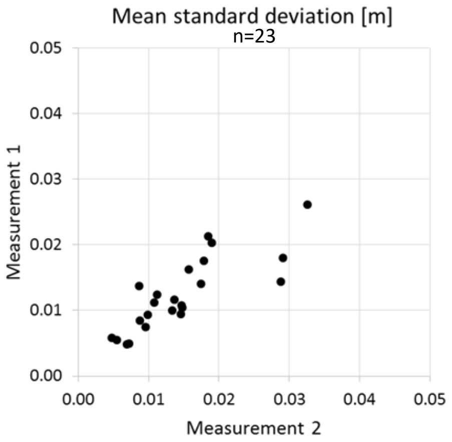

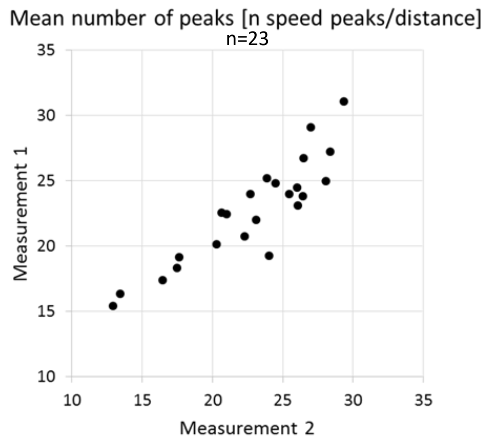

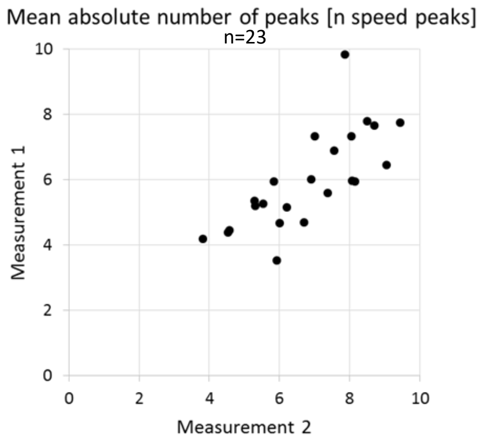

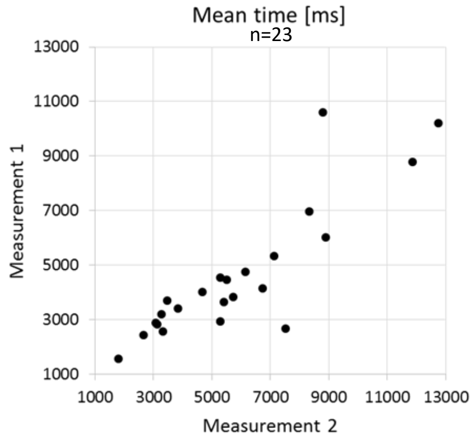

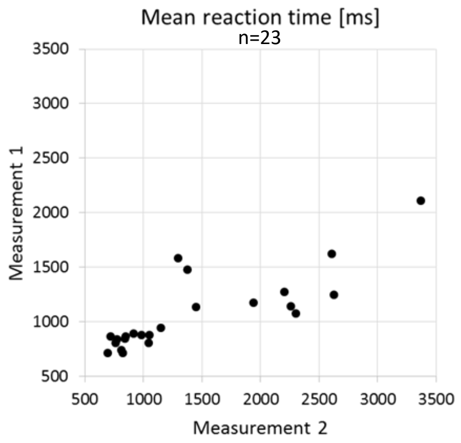

Displayed are the data of each parameter the Quality of Movement (QoM) assessment (means of three trials): The mean distance-to-path-ratio (unitless), the mean standard deviation on the target in meters (m), the mean number of speed peaks per covered distance (n speed peaks/distance), the mean absolute number of speed peaks (n speed peaks), the mean time needed to reach a target, in milliseconds (ms) and the mean reaction time in milliseconds (ms). The X-axis represents the second measurement, the Y-axis represents the first measurement.
