# Supplementary material for: Test–retest reliability of upper limb robotic exoskeleton assessments in children and youths with brain lesions
Source: Sci Rep. 2022 Oct 6;12:16685. doi: 10.1038/s41598-022-20588-8 (PMC9537308; doi:10.1038/s41598-022-20588-8)
Supplement: Supplementary file 5 — Supplementary Information 5. [file 41598_2022_20588_MOESM5_ESM.pdf]

Supplementary information file 5
Distribution of the data of each parameter obtained from the Circle assessment

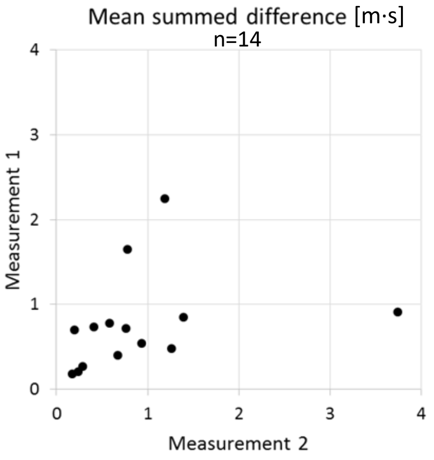

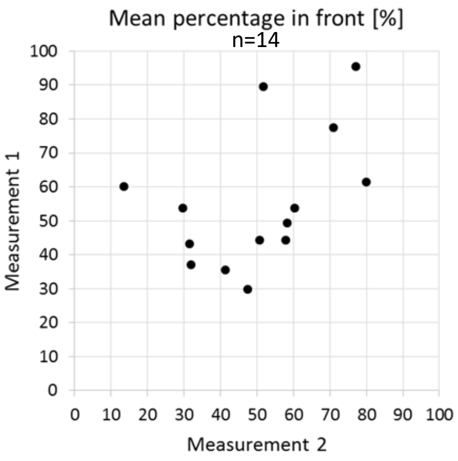

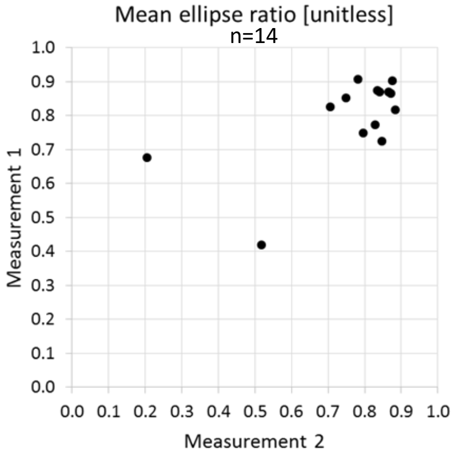

Displayed are the data of each parameter the Circle assessment (means of three trials): Mean summed difference between the current position and the reference circle in meters (m), mean percentage of the tie the participant was in front of the reference circle in meters (m), and the mean ellipse ratio between the minimal and the maximal radius of the least-squares fitted ellipse (unitless). The X-axis represents the second measurement, the Y-axis represents the first measurement.
