# Supplementary material for: Test–retest reliability of upper limb robotic exoskeleton assessments in children and youths with brain lesions
Source: Sci Rep. 2022 Oct 6;12:16685. doi: 10.1038/s41598-022-20588-8 (PMC9537308; doi:10.1038/s41598-022-20588-8)
Supplement: Supplementary file 6 — Supplementary Information 6. [file 41598_2022_20588_MOESM6_ESM.pdf]

Supplementary information file 6

Distribution of the data of each parameter obtained from the Workspace assessment

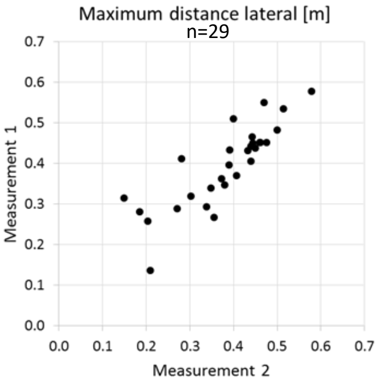

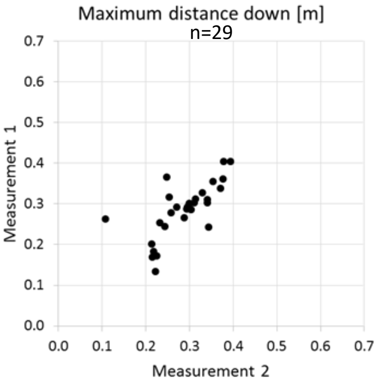

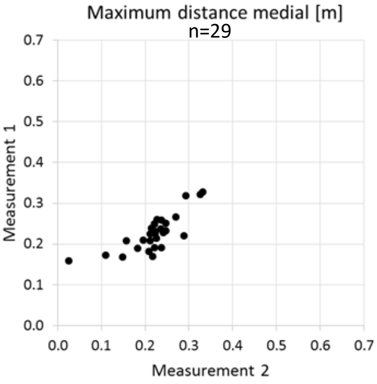

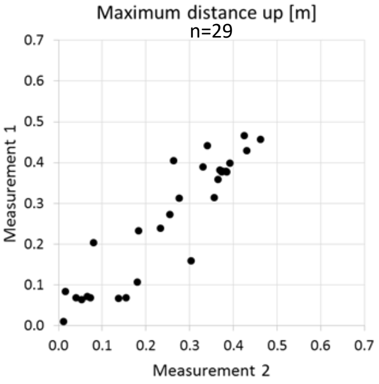

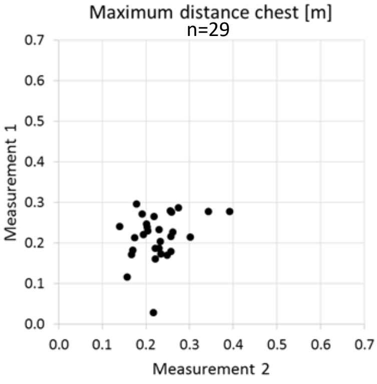

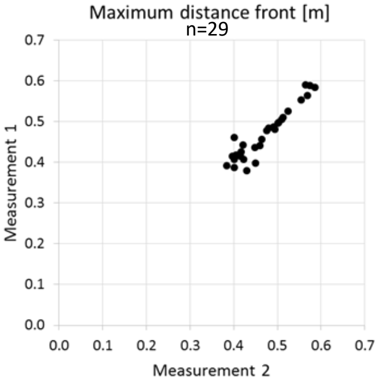

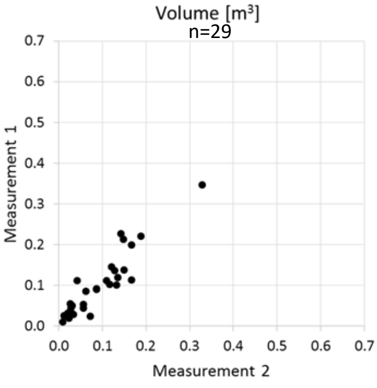

Displayed are the absolute data of each parameter the Workspace assessment: maximum distance in medial direction, lateral direction, down, up, to the front and to the chest in meters (m) and the maximum reachable volume in cubic meters (m^3). The X-axis represents the second measurement, the Y-axis represents the first measurement.
